# Supplementary figures and images for: Training and testing of a gradient boosted machine learning model to predict adverse outcome in patients presenting to emergency departments with suspected covid-19 infection in a middle-income setting
Source: PLOS Digit Health. 2023 Sep 20;2(9):e0000309. doi: 10.1371/journal.pdig.0000309 (PMC10511129; doi:10.1371/journal.pdig.0000309)

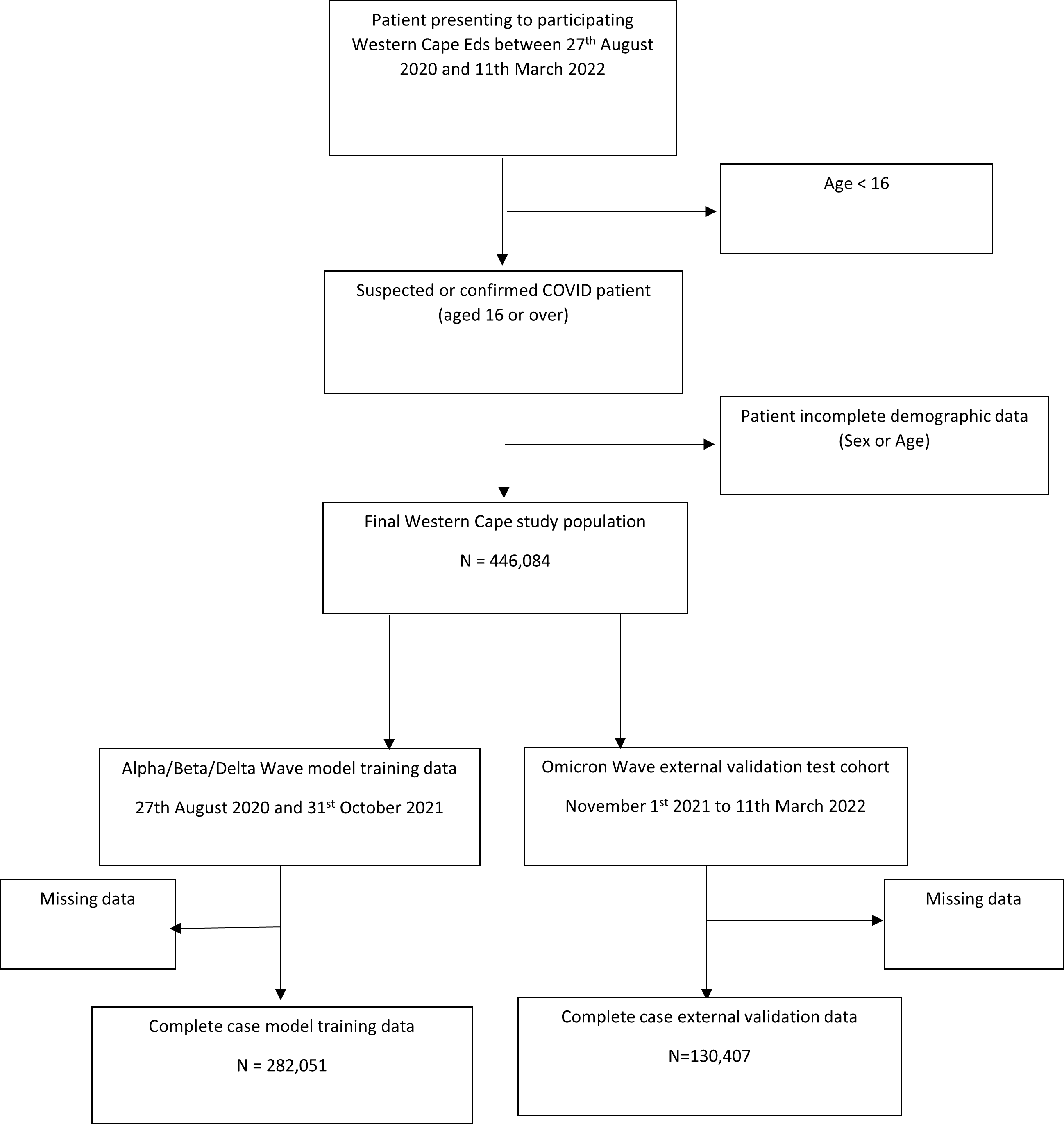

Supplement: S1 Fig — (TIF) [file pdig.0000309.s001.tif]

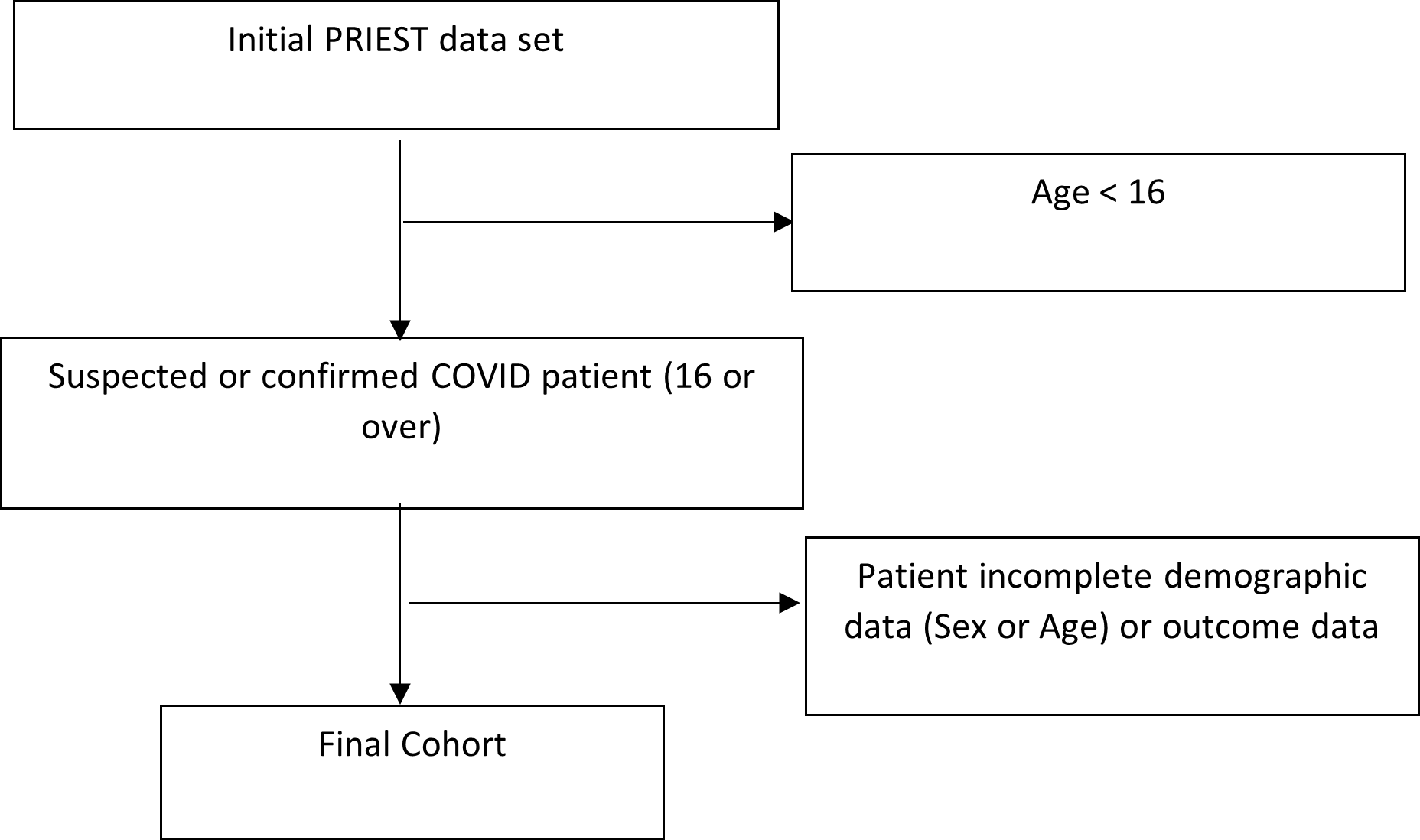

Supplement: S2 Fig — (TIF) [file pdig.0000309.s002.tif]

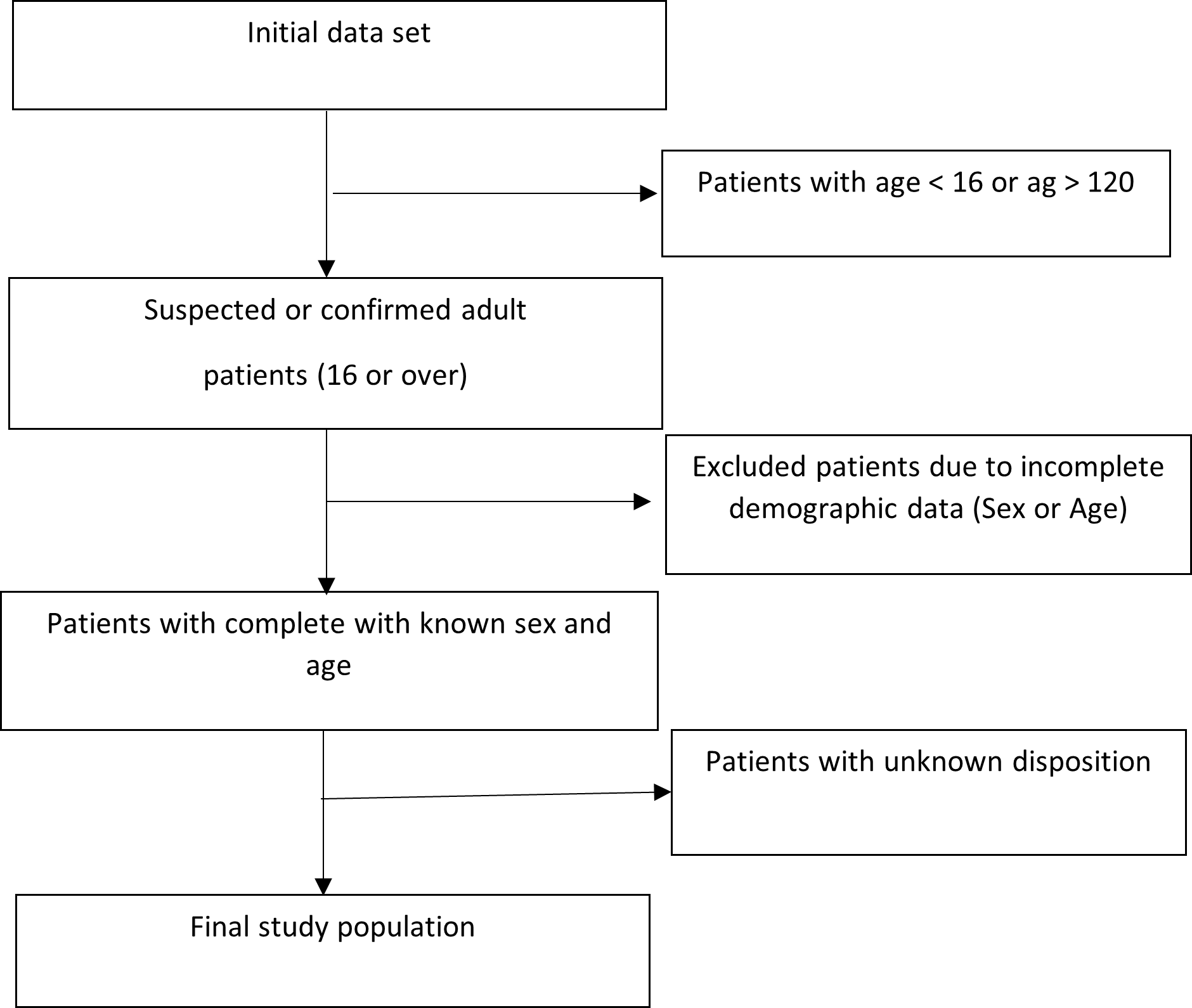

Supplement: S3 Fig — (TIF) [file pdig.0000309.s003.tif]

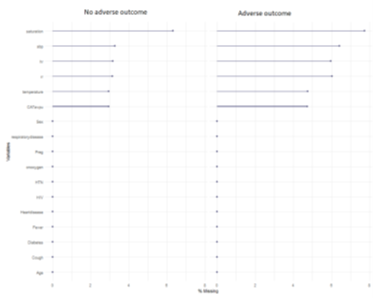

Supplement: S4 Fig — (TIF) [file pdig.0000309.s004.tif]

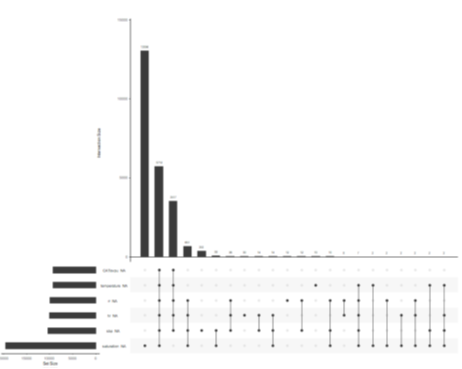

Supplement: S5 Fig — N = 305,564. 7.7% of cases (n = 23,513) had missing data. (TIF) [file pdig.0000309.s005.tif]

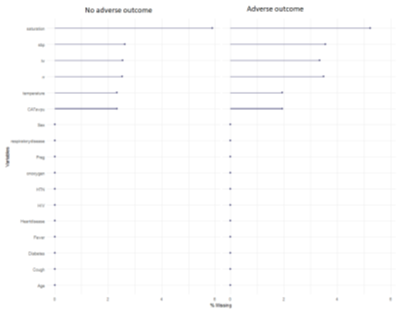

Supplement: S6 Fig — (TIF) [file pdig.0000309.s006.tif]

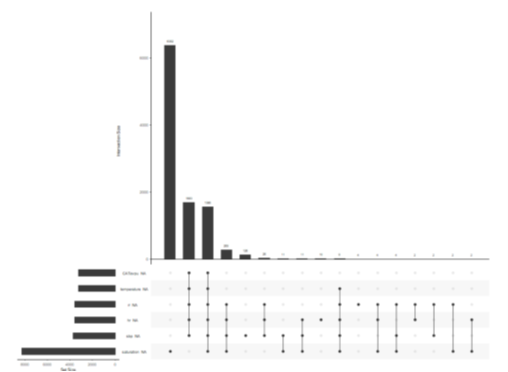

Supplement: S7 Fig — N = 140,520. 7.2% of cases (n = 10,113) had missing data. (TIF) [file pdig.0000309.s007.tif]

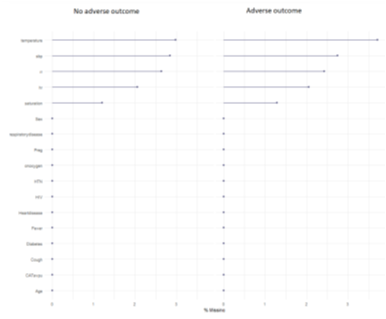

Supplement: S8 Fig — (TIF) [file pdig.0000309.s008.tif]

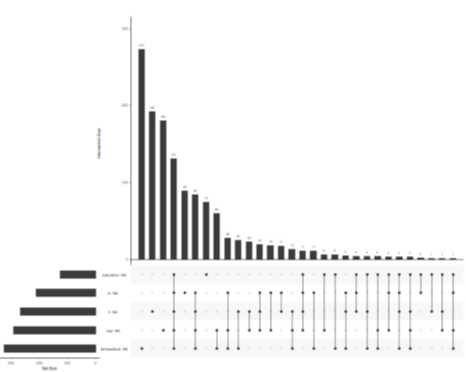

Supplement: S9 Fig — N = 20,698. 6.2% of cases (n = 1,291) had missing data. (TIF) [file pdig.0000309.s009.tif]

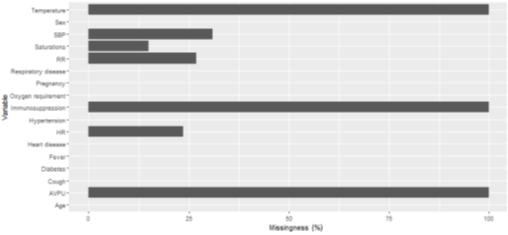

Supplement: S10 Fig — (TIF) [file pdig.0000309.s010.tif]
