# Supplementary material for: Training and testing of a gradient boosted machine learning model to predict adverse outcome in patients presenting to emergency departments with suspected covid-19 infection in a middle-income setting
Source: PLOS Digit Health. 2023 Sep 20;2(9):e0000309. doi: 10.1371/journal.pdig.0000309 (PMC10511129; doi:10.1371/journal.pdig.0000309)
Supplement: S1 Text — (DOCX) [file pdig.0000309.s017.docx]

**S1 Table. Characteristics of Western Cape Omicron wave test cohort**

| **Characteristic** | **Statistic/level** | **Adverse outcome** | **No adverse outcome** | **Total** |
| --- | --- | --- | --- | --- |
|  | N | 2,787 (2%) | 137,733 (98%) | 140,520 |
| Age (years) | Mean (SD) | 48.8 (18.8) | 43.2 (17.1) | 43.3 (17.2) |
|  | Median (IQR) | 46 (33, 64) | 40 (29, 56) | 41 (29, 56) |
|  | Range | 16 to 95 | 16 to 110 | 16 to 110 |
| Sex | Male | 1,658 (59.5%) | 70,056 (50.9%) | 71,714 (51%) |
|  | Female | 1,129 (40.5%) | 67,677 (49.1%) | 68,806 (49%) |
| Comorbidities | Asthma/COPD | 474 (17%) | 20,755 (15.1%) | 21,229 (15.1%) |
|  | Other Chronic respiratory disease | 7 (0.3%) | 297 (0.2%) | 304 (0.2%) |
|  | Diabetes | 801 (28.7%) | 21,307 (15.5%) | 22,108 (15.7%) |
|  | Hypertension | 1,008 (36.2%) | 36,227 (26.3%) | 37,235 (26.5%) |
|  | Immunosuppression (HIV) | 488 (17.5%) | 24,430 (17.7%) | 24,918 (17.7%) |
|  | Heart Disease | 912 (32.7%) | 24,078 (17.5%) | 24,990 (17.8%) |
|  | Pregnant | 43 (1.5%) | 727 (0.5%) | 770 (0.6%) |
| AVPU | Missing |  |  | 3,249 (2.3%) |
|  | Alert | 1,702 (61.1%) | 125,337 (91%) | 127,039 (90.4%) |
|  | Voice | 96 (3.4%) | 1,916 (1.4%) | 2,012 (1.4%) |
|  | Confused | 180 (6.5%) | 5,327 (3.9%) | 5,057 (3.9%) |
|  | Pain | 201 (7.2%) | 975 (0.7%) | 1,176 (0.8%) |
|  | Unresponsive | 554 (19.9%) | 983 (0.7%) | 1,537 (2.3%) |
| Systolic BP (mmHg) | Missing |  |  | 3,707 (2.6%) |
|  | N | 2,688 | 134,125 | 136,813 |
|  | Mean (SD) | 126 (30) | 129.7 (25.3) | 129.6 (25.4) |
|  | Median (IQR) | 122 (107,143) | 127 (113,143) | 127 (113,143) |
|  | Range | 52 to 288 | 50 to 300 | 50 to 300 |
| Pulse rate (beats/min) | Missing |  |  | 3,582 (2.6%) |
|  | N | 2,694 | 134,244 | 136,938 |
|  | Mean (SD) | 100 (24.7) | 93 (21.1) | 93.1 (21.2) |
|  | Median (IQR) | 99 (83,115) | 92 (78, 106) | 92 (78,106) |
|  | Range | 12 to 300 | 10 to 300 | 10 to 300 |
| Respiratory rate (breaths/min) | Missing |  |  | 3,571 (2.5%) |
|  | N | 2,690 | 134,259 | 136,949 |
|  | Mean (SD) | 19 (5.5) | 18.1 (3.6) | 18.1 (3.6) |
|  | Median (IQR) | 19 (17,22) | 18 (16,20) | 18 (16,20) |
|  | Range | 8 to 60 | 1 to 60 | 1 to 60 |
| Oxygen saturation | Missing |  |  | 8,260 (5.9%) |
|  | N | 2,641 | 129,619 | 132,260 |
|  | Mean (SD) | 93.9 (8.1) | 96.3 (4.9) | 96.2 (5) |
|  | Median (IQR) | 96 (93, 99) | 97 (96, 99) | 97 (95, 99) |
|  | Range | 13 to 100 | 10 to 100 | 10 to 100 |
| Oxygen administration | Missing |  |  | 7,910 (5.6%) |
|  | N | 2,662 | 129,948 | 132,610 |
|  | 1 (air) | 1,516 (57%) | 123,044 (94.7%) | 124,560 (93.9%) |
|  | 2 (40% O2) | 58 (2.2%) | 2,407 (1.9%) | 2,465 (1.9%) |
|  | 3 (28% O2) | 2 (0.1%) | 82 (0.1%) | 84 (0.1%) |
|  | 4 (Nasal prongs) | 1,19 (4.5%) | 2,610 (2%) | 2,729 (2.1%) |
|  | 5 (FM neb) | 11 (0.4%) | 378 (0.3%) | 389 (0.3%) |
|  | 6 (rebreather mask) | 1,10 (4.1%) | 1,315 (1%) | 1,425 (1.1%) |
|  | 7 (nasal prongs and rebreather mask) | 17 (0.6%) | 112 (0.1%) | 1,29 (0.1%) |
|  | 8 intubated | 776 (29.2%) | 0 | 776 (0.6%) |
|  | 9 NIV | 53 (2%) | 0 | 53 (0.04%) |
| Temperature (°C) | Missing |  |  | 3,258 (2.3%) |
|  | N | 2,733 | 134,529 | 137,262 |
|  | Mean (SD) | 36.3 (1.1) | 36.3 (0.7) | 36.3 (0.7) |
|  | Median (IQR) | 36.3 (35.9, 36.7) | 36.3 (36, 36.6) | 36.3 (36, 36.6) |
|  | Range | 25 to 40 | 25 to 41.9 | 25 to 41.9 |
| Cough | Missing |  |  | 93,962 (30.8%) |
|  | Present | 80 (2.9%) | 3,500 (2.5%) | 3,580 (2.6%) |
| Fever | Missing |  |  | 41,524 (29.6%) |
|  | Present | 25 (0.9%) | 1,169 (0.9%) | 1,194 (0.9%) |
| COVID PCR | Positive | 2,119 (76%) | 26,485 (19.2%) | 28,604 (20.4%) |
| Hospital admission | ICU | 6,77 (24.3%) | 0 | 6,77 (0.5%) |
| Death | Within 30 days contact | 1,431 (51.4%) | 0 | 1,431 (1%) |
