# Supplementary material for: Training and testing of a gradient boosted machine learning model to predict adverse outcome in patients presenting to emergency departments with suspected covid-19 infection in a middle-income setting
Source: PLOS Digit Health. 2023 Sep 20;2(9):e0000309. doi: 10.1371/journal.pdig.0000309 (PMC10511129; doi:10.1371/journal.pdig.0000309)
Supplement: S2 Text — (DOCX) [file pdig.0000309.s018.docx]

**S2 Table. Population characteristics UK PRIEST test cohort**

| **Characteristic** | **Statistic/level** | **Adverse outcome** | **No adverse outcome** | **Total** |
| --- | --- | --- | --- | --- |
|  | N | 4,579 (22.1%) | 16,119 (77.9%) | 20,698 |
| Age (years)* | 16-49 | 369 (8.1%) | 5,256 (32.6%) | 5,625 (27.2%) |
|  | 50-65 | 981 (21.4%) | 4,186 (26%) | 5,167 (25%) |
|  | 66-80 | 1,527 (33.4%) | 3,727 (23.1%) | 5,254 (25.4%) |
|  | >80 | 1,702 (37.2%) | 2,950 (18.3%) | 4,652 (22.5%) |
| Sex | Male | 2,661 (58.1%) | 7,540 (46.8%) | 10,201 (49.3%) |
|  | Female | 1,918 (41.9%) | 8,579 (53.2%) | 10,497 (50.7%) |
| Comorbidities | Asthma | 556 (12.1%) | 2,820 (17.5%) | 3,376 (16.3%) |
|  | Other Chronic respiratory disease | 1,045 (22.8%) | 2,693 (16.7%) | 3,738 (18.1%) |
|  | Diabetes | 1,274 (27.8%) | 2,816 (17.5%) | 4,090 (19.8%) |
|  | Hypertension | 1,828 (39.9%) | 4,538 (28.2%) | 6,366 (30.8%) |
|  | Immunosuppression | 171 (3.7%) | 456 (2.8%) | 627 (3%) |
|  | Heart Disease | 912 (32.7%) | 24,078 (17.5%) | 4,661 (22.5%) |
|  | Pregnant | 6 (0.1%) | 79 (0.5%) | 85 (0.4%) |
| AVPU | Missing |  |  | 2,063 (10%) |
|  | Alert | 3,030 (66.2%) | 13,335 (82.9%) | 16,385 (79.2%) |
|  | Voice | 263 (5.7%) | 234 (1.5%) | 497 (2.4%) |
|  | Confused | 557 (12.2%) | 907 (5.6%) | 1,464 (7.1) |
|  | Pain | 114 (2.5%) | 65 (0.4%) | 179 (0.9%) |
|  | Unresponsive | 77 (1.7%) | 33 (0.2%) | 1,10 (0.6%) |
| Systolic BP (mmHg) | Missing |  |  | 585 (2.8%) |
|  | N | 4,453 | 15,660 | 20,113 |
|  | Mean (SD) | 130.1 (26.7) | 135.9 (24.2) | 134.6 (24.9) |
|  | Median (IQR) | 129 (112,147) | 134 (120, 150) | 133 (118, 149) |
|  | Range | 47 to 254 | 37 to 264 | 37 to 264 |
| Pulse rate (beats/min) | Missing |  |  | 426 (2.1%) |
|  | N | 4,485 | 15,787 | 20,272 |
|  | Mean (SD) | 98.3 (23.3) | 93.9 (20.9) | 94.9 (21.5) |
|  | Median (IQR) | 97 (83,112) | 92 (80, 107) | 93 (80, 108) |
|  | Range | 8 to 209 | 11 to 220 | 11 to 220 |
| Respiratory rate (breaths/min) | Missing |  |  | 536 (2.6%) |
|  | N | 4,468 | 15,694 | 20,162 |
|  | Mean (SD) | 27.1 (8.5) | 22.2 (6.1) | 23.3 (7) |
|  | Median (IQR) | 25 (21,32) | 20 (18,24) | 22 (18,26) |
|  | Range | 6 to 99 | 5 to 99 | 5 to 99 |
| Oxygen saturation | Missing |  |  | 254 (1.2%) |
|  | N | 4,520 | 15,924 | 20,444 |
|  | Mean (SD) | 91.5 (8.8) | 95.6 (5.8) | 94.7 (6.8) |
|  | Median (IQR) | 84 (89, 96) | 97 (95, 98) | 96 (94, 98) |
|  | Range | 22 to 100 | 13 to 100 | 13 to 100 |
| Oxygen administration | On Oxygen | 1,964 (42.9%) | 2,249 (14%) | 4,213 (20.4%) |
| Temperature (°C) | Missing |  |  | 651 (2.3%) |
|  | N | 4,409 | 15,638 | 20,047 |
|  | Mean (SD) | 37.3 (1.2) | 37.1 (1) | 37.1 (1.1) |
|  | Median (IQR) | 37.2 (36.5, 38.2) | 36.9 (36.4, 37.7) | 37 (36.4, 37.8) |
|  | Range | 31.3 to 41.3 | 25.9 to 42.1 | 25.9 to 42.1 |
| Cough | Present | 2,659 (58.1%) | 10,211 (63.4%) | 12,870 (62.2%) |
| Fever | Present | 2,271 (49.6%) | 7,916 (49.1%) | 10,187 (49.2%) |
| Clinical impression | COVID | 3,419 (77.9%) | 10,518 (68.5%) | 13,937 (70.6%) |
| Organ Support | Any | 2,046 (44.7%) | 0 | 2,046 (9.9%) |
| Death | Within 30 days contact | 3,222 (70.4%) | 0 | 3,222 (15.6%) |
