# Supplementary material for: Training and testing of a gradient boosted machine learning model to predict adverse outcome in patients presenting to emergency departments with suspected covid-19 infection in a middle-income setting
Source: PLOS Digit Health. 2023 Sep 20;2(9):e0000309. doi: 10.1371/journal.pdig.0000309 (PMC10511129; doi:10.1371/journal.pdig.0000309)
Supplement: S3 Text — (DOCX) [file pdig.0000309.s019.docx]

**S3 Table. Population characteristics Sudan test cohort**

| **Characteristic** | **Statistic/level** | **Adverse outcome** | **No adverse outcome** | **Total** |
| --- | --- | --- | --- | --- |
|  | N | 874 | 1,709 | 2,583 |
| Age (years) | Mean (SD) | 65.8 (13.7) | 62.8 (14.1) | 63.8 (14) |
|  | Median (IQR) | 68 (59) | 65 (55) | 65 (55) |
|  | Range | 17-99 | 16-100 | 16 to 100 |
| Sex | Male | 586 | 1089 | 1675(64.8%) |
|  | Female | 288 | 620 | 908(35.2%) |
| Comorbidities | Asthma | 2 | 4 | 6(0.2%) |
|  | Cardiovascular disease | 13 | 20 | 33(1.3%) |
|  | Diabetes | 14 | 36 | 50 (1.9%) |
|  | Hypertension | 122 | 209 | 331 (12.8%) |
|  | Malignancy | 1 | 3 | 4 (0.2%) |
|  | Renal impairment | 10 | 21 | 31 (1.2%) |
| Symptoms | Cough | 35 | 81 | 116 (4.5%) |
| Systolic BP (mmHg) | Missing | - | - | 797 (30.9%) |
|  | N | 588 | 1198 | 1786 (69.1%) |
|  | Mean (SD) | 128.7 (26.2) | 131.5 (22.5) | 130.6 (23.8) |
|  | Median (IQR) | 127 (112) | 130 (118) | 130 (155) |
|  | Range | 53 to 257 | 60 to 245 | 53 to 257 |
| Heart rate | Missing | - | - | 610 (23.6%) |
|  | N | 688 | 1285 | 1973 (76.4% |
|  | Mean (SD) | 97.5(21.4) | 91.3(18.4) | 93.5 (19.7) |
|  | Median (IQR) | 97 (84) | 90 (79) | 92 (80) |
|  | Range | 28 to 196 | 12 to 155 | 12 to 196 |
| RR | Missing | - | - | 694 (26.9%) |
|  | N | 661 | 1228 | 1889 (73.1%) |
|  | Mean (SD) | 33.5 (10.2) | 29.8 (9.3) | 31 (9.8) |
|  | Median (IQR) | 32 (26) | 28 (24) | 29 (25) |
|  | Range | 12 to 88 | 10 to 108 | 10 to 108 |
| Short of breath | N | 32 | 74 | 106 (4.1%) |
| Oxygen Saturation | Missing | - | - | 385 (14.9%) |
|  | N | 782 | 1416 | 2198 (85.1%) |
|  | Mean (SD) | 83.3 (17) | 84.3 (14.4) | 84 (15.4) |
|  | Median (IQR) | 89.5 (77) | 88 (80) | 88 (79) |
|  | Range | 18 to 100 | 10 to 100 | 10 to 100 |
| Supplemental Oxygen | Missing | - | - | 670 (25.9%) |
|  | Non-rebreathe Mask | 222 | 542 | 764 (29.6%) |
|  | Room air | 150 | 522 | 672 (26%) |
|  | Non-invasive ventilation | 298 | 0 | 298 (11.5%) |
|  | Nasal Cannula | 8 | 71 | 79 (3.1%) |
|  | Dual flow (e.g. nasal cannula and oxygen mask) | 41 | 56 | 97 (3.8%) |
|  | Intubated | 2 | 0 | 2 (0.1%) |
|  | Simple Face Mask | 1 | 0 | 1 (0.0%) |
| Disposition | transferred | 221 | 1255 | 1476 (57.1%) |
|  | deceased | 596 | 0 | 596 (23.1%) |
|  | discharged | 51 | 363 | 414 (16.0%) |
|  | admitted | 6 | 91 | 97 (3.8%) |
| Swab result | Missing |  |  | 1266 (49.0%) |
|  | Positive | 306 | 689 | 995 (38.5%) |
|  | Negative | 81 | 172 | 253 (9.8%) |
|  | No results | 34 | 35 | 69 (2.7%) |
| Level of care | Missing | 719 | 1055 | 1774 (68.7%) |
|  | Ward | 96 | 654 | 750 (29%) |
|  | HDU | 35 | 0 | 35 (1.3%) |
|  | ICU | 24 | 0 | 24 (0.9%) |
