# Supplementary material for: Training and testing of a gradient boosted machine learning model to predict adverse outcome in patients presenting to emergency departments with suspected covid-19 infection in a middle-income setting
Source: PLOS Digit Health. 2023 Sep 20;2(9):e0000309. doi: 10.1371/journal.pdig.0000309 (PMC10511129; doi:10.1371/journal.pdig.0000309)
Supplement: S4 Text — (DOCX) [file pdig.0000309.s020.docx]

**S4 Table. Diagnostic accuracy at different base case model thresholds in Western Cape Alpha/Beta/Delta wave training data**

| Cut-point (%) | Sensitivity | Specificity | Correctly classified | LR+ | LR- |
| --- | --- | --- | --- | --- | --- |
| >0 | 100.0% | 0.0% | 4.1% | 1.0 | - |
| >=10 | 82.1% | 82.7% | 82.7% | 4.7 | 0.2 |
| >=20 | 55.3% | 95.8% | 94.1% | 13.2 | 0.5 |
| >=30 | 38.2% | 98.5% | 96.0% | 25.0 | 0.6 |
| >=40 | 28.0% | 99.3% | 96.4% | 39.5 | 0.7 |
| >=50 | 21.0% | 99.7% | 96.4% | 61.2 | 0.8 |
| >=60 | 15.7% | 99.8% | 96.4% | 95.5 | 0.8 |
| >=70 | 9.8% | 99.9% | 96.2% | 146.4 | 0.9 |
| >=80 | 5.6% | 100.0% | 96.1% | 169.0 | 0.9 |
| >=90 | 3.3% | 100.0% | 96.0% | 596.1 | 1.0 |
| 100 | 0.0% | 100.0% | 95.9% | - | 1.0 |
