# Supplementary material for: Training and testing of a gradient boosted machine learning model to predict adverse outcome in patients presenting to emergency departments with suspected covid-19 infection in a middle-income setting
Source: PLOS Digit Health. 2023 Sep 20;2(9):e0000309. doi: 10.1371/journal.pdig.0000309 (PMC10511129; doi:10.1371/journal.pdig.0000309)
Supplement: S5 Text — (DOCX) [file pdig.0000309.s021.docx]

**S5 Table. Diagnostic accuracy at different base case model thresholds in thresholds in Western Cape Omicron wave test data**

| Cut-point (%) | Sensitivity | Specificity | Correctly classified | LR+ | LR- |
| --- | --- | --- | --- | --- | --- |
| >0 | 100.0% | 0.0% | 2.0% | 1.0 |  |
| >=10 | 68.4% | 84.7% | 84.4% | 4.5 | 0.4 |
| >=20 | 37.8% | 96.6% | 95.4% | 11.1 | 0.6 |
| >=30 | 29.0% | 98.8% | 97.4% | 24.8 | 0.7 |
| >=40 | 23.1% | 99.5% | 98.0% | 45.3 | 0.8 |
| >=50 | 19.0% | 99.8% | 98.2% | 78.9 | 0.8 |
| >=60 | 16.4% | 99.9% | 98.2% | 137.5 | 0.8 |
| >=70 | 13.8% | 99.9% | 98.2% | 219.9 | 0.9 |
| >=80 | 9.7% | 100.0% | 98.2% | 275.6 | 0.9 |
| >=90 | 5.8% | 100.0% | 98.1% | 529.2 | 0.9 |
| 100 | 0.0% | 100.0% | 98.0% |  | 1.0 |
