# Supplementary material for: Training and testing of a gradient boosted machine learning model to predict adverse outcome in patients presenting to emergency departments with suspected covid-19 infection in a middle-income setting
Source: PLOS Digit Health. 2023 Sep 20;2(9):e0000309. doi: 10.1371/journal.pdig.0000309 (PMC10511129; doi:10.1371/journal.pdig.0000309)
Supplement: S6 Text — (DOCX) [file pdig.0000309.s022.docx]

**S6 Table. Diagnostic accuracy at different base case model thresholds in PRIEST test data**

| Cut-point (%) | Sensitivity | Specificity | Correctly classified | LR+ | LR- |
| --- | --- | --- | --- | --- | --- |
| >0 | 100.0% | 0.0% | 21.9% | 1.0 | - |
| >=10 | 91.7% | 31.1% | 44.4% | 1.3 | 0.3 |
| >=20 | 64.0% | 67.9% | 67.0% | 2.0 | 0.5 |
| >=30 | 37.7% | 87.5% | 76.6% | 3.0 | 0.7 |
| >=40 | 21.3% | 95.0% | 78.9% | 4.3 | 0.8 |
| >=50 | 11.8% | 98.0% | 79.1% | 6.0 | 0.9 |
| >=60 | 5.3% | 99.3% | 78.7% | 7.2 | 1.0 |
| >=70 | 1.5% | 99.8% | 78.3% | 8.2 | 1.0 |
| >=80 | 0.2% | 100.0% | 78.1% | 7.1 | 1.0 |
| >=90 | 0.0% | 100.0% | 78.1% | 3.6 | 1.0 |
| 100 | 0.0% | 100.0% | 78.1% | - | 1.0 |
