# Supplementary material for: Training and testing of a gradient boosted machine learning model to predict adverse outcome in patients presenting to emergency departments with suspected covid-19 infection in a middle-income setting
Source: PLOS Digit Health. 2023 Sep 20;2(9):e0000309. doi: 10.1371/journal.pdig.0000309 (PMC10511129; doi:10.1371/journal.pdig.0000309)
Supplement: S7 Text — (DOCX) [file pdig.0000309.s023.docx]

**S7 Table. Diagnostic accuracy at different base case model thresholds in thresholds in Sudanes test data**

| Cut-point (%) | Sensitivity | Specificity | Correctly classified | LR+ | LR- |
| --- | --- | --- | --- | --- | --- |
| >0 | 100.0% | 0.0% | 35.1% | 1.0 |  |
| >=10 | 97.6% | 9.1% | 40.2% | 1.1 | 0.3 |
| >=20 | 73.7% | 43.7% | 54.3% | 1.3 | 0.6 |
| >=30 | 35.5% | 75.5% | 61.5% | 1.5 | 0.9 |
| >=40 | 21.2% | 85.7% | 63.0% | 1.5 | 0.9 |
| >=50 | 9.3% | 95.9% | 65.5% | 2.3 | 0.9 |
| >=60 | 5.3% | 97.9% | 65.4% | 2.5 | 1.0 |
| >=70 | 1.3% | 99.5% | 65.0% | 2.8 | 1.0 |
| >=80 | 0.0% | 99.9% | 64.8% | 0.0 | 1.0 |
| >80 | 0.0% | 100.0% | 64.9% |  | 1.0 |
