# Supplementary material for: Training and testing of a gradient boosted machine learning model to predict adverse outcome in patients presenting to emergency departments with suspected covid-19 infection in a middle-income setting
Source: PLOS Digit Health. 2023 Sep 20;2(9):e0000309. doi: 10.1371/journal.pdig.0000309 (PMC10511129; doi:10.1371/journal.pdig.0000309)
Supplement: Attachment — Submitted filename: Response to peer review.docx [file pdig.0000309.s024.docx]

**Training and testing of a gradient boosted machine learning model to predict adverse outcome in patients presenting to emergency departments with suspected COVID-19 infection in a middle-income setting**

**Response to peer review**

Thank you for the opportunity to make corrections to our manuscript. We are grateful for the referees' helpful comments to improve our paper. We have responded to each suggestion below and attach a marked manuscript with our edits highlighted in red text. We hope our manuscript is now ready for publication.

**General comments to Author**

We are pleased that the reviewers felt this study met PLOS Digital Health’s publication criteria, the statistical analysis was appropriate and rigorous, and the manuscript was presented in an intelligible fashion.

**Specific comments to Author**

**Review 1**

- *It could use some very minor proofreading including what is meant by ‘retrospective’ cohort.*

Thank you for this comment. We have re-read the manuscript to detect any typographical errors and have clarified our definition of retrospective cohort.

- *It may be worthwhile stating why some other machine learning baselines were not performed.*

Thank you for raising this point. We have edited the methods to further highlight the rationale for choosing the XGBoost machine learning algorithm. Logistic regression modelling was performed in another sub-study which has been published elsewhere. We have highlighted this paper in the discussion and provided a reference.

**Review 2**

- *It is not clear why the train/test split was based on temporality. The model could have been more accurate if it included representations from all variants and times.*

Thank you for raising this important issue. The overall aim of the study was to develop a machine learning algorithm for use in middle income settings, such as the Western Cape in South Africa. As Omicron is the current dominant variant and the later time is most applicable to the current population requiring risk-stratification in an ED setting in the Western Cape and other LMICs, we pulled out this period to allow estimates to be as applicable as possible to the current stage of the pandemic.

However, we agree that a model trained on all time points might be more accurate and would give an indication of likely performance in future waves, irrespective of the specifics of vaccination prevalence and variant dominance. We have therefore included an additional analysis where the model is trained on Western Cape data across the alpha, beta, delta, and omicron waves, with a random train/test split. We have retained the PRIEST (ancestral variant) and Sudanese (ancestral and Eta variant) data as external validation / separate test populations. Inclusion of such patients in the training data would not fit with our objective of developing a model for the South African middle-income setting. These separate external validations also provide insights into transportability of the model as discussed further below.

- *It is unfair to expect that the model will perform across external validation sets. Such a lack of generalisation is not really overfitting, but it is because of the disparity between train and test sets.*

Thank you for emphasising this point. We agree that internal validation focuses on reproducibility and overfitting, while external validation focuses on transportability. The purpose of assessing performance across different locations and over time is to maximize our understanding of model transportability. We have edited the discussion to address this issue and highlight potential differences in patient populations and measurements of predictors and outcomes.

- *It seems the data got split roughly to a 2:1 proportion. With a large dataset at hand the authors should have chosen a larger training set.*

Thank you for this suggestion. In the original analysis we split the Western Cape data into alpha/beta/delta (training data) and omicron (test data) wave periods, which gave a 2:1 split. In the additional analysis including all Western Cape patients we have retrained the model using an 80:20 random split across all variant waves. As noted, this should allow the model to learn from a larger variety of clinical presentations.

- *My real difficulty is with the clinical value of this model. A model based on less representativeness of training, less accuracy in external settings and less acceptability in clinical practice seems to be only an academic exercise.*

Thank you for recognising this concern. It is important that internally valid and generalisable research is translated into clinical practice when appropriate. The Western Cape emergency medical services use an electronic patient record with the functionality to incorporate electronic decision support. Machine learning algorithms can also be deployed as mobile phone applications. There is consequently the potential for clinical use of our model. However, to provide a well rounded discussion we also present a transparent discussion of the challenges and barriers of using machine learning models in emergency care. We have edited our discussion to reflect these points.
